# The Common Accidents of Every-Day Life

**Published:** 1887-02-05

**Authors:** A. W. Hare

**Affiliations:** Senior Demonstrator of Surgery in the University of Edinburgh


### The Common Accidents of Every-day Life.

By A. W. Hare, M.B., F.R.C.S.E.,
Senior Demonstrator of Surgery in the University of Edinburgh.

[Dr. Hare will be glad to receive instances of common accidents, and to answer inquiries addressed to him, care of the Editor of The Hospital.]

### EARLY MISADVENTURES.

### I.—Dangers Connected with Swallowing.

(*Continued from p.* 289.)

**Case illustrating the dangers of choking.** Since the appearance of the preceding part of this paper the following distressing case has been communicated to the writer. A fine healthy boy, eleven months old, was creeping about and amusing himself on a soft rug on the floor of his nursery,—such a rug forms a very suitable playground for children at that age. The smooth glass stopper of a decanter was given him to play with and roll about. Suddenly his warblings of delight changed to a sharp cry of pain, which was followed by coughing and choking. His mother hastened to help him, but was unable to detect the cause of his great distress, and vainly endeavoured to afford relief. The doctor was sent for, but before his arrival life was extinct. A spicule of glass had been chipped off the stopper, and, becoming fixed in the throat, had caused fatal choking. Such an incident carries with it its own lesson; if any comment were added it would be that a young healthy life, with all its possibilities of good, the sunshine of a household, was lost *for lack of a little simple knowledge.*

(*b*) **Poisoning in Infants.** The other chief danger connected with swallowing is that the infant may obtain in this way some poisonous substance. These cases differ from similar occurrences in after life, from the fact that substances known to be poisonous never come within reach of an infant. The sources of danger are articles supposed to be harmless, which, however, contain poisonous matter that can be extracted and swallowed. The commonest sources are textures containing a soluble dye-stuff, such as bright red, or green woollen materials which may contain arsenic in a form that can be dissolved in the infant's mouth. Or poison may be given unwittingly by ignorant persons, as in a recent case where a number of children, including an infant, were poisoned by yew berries, the older children having shared the deadly feast with the younger. Under this head also must be noticed those cases, sadly frequent in great cities, where raw spirit is given to infants by ignorant or inebriated parents, in not a few of which immediate death has taken place. In all such cases of poisoning, a doctor must be summoned at once. But immediate treatment should be carried out by those in charge. The symptoms will vary according to the poison that has been taken. In all cases, however, where there is evidence of some such substance having been swallowed, two things must be done. First, dilute the poison in the stomach with warm water; second, empty the stomach by causing vomiting. Fill the infant's bottle with warm water, and get as much of it swallowed as possible. This is not difficult if the lips be judiciously stimulated by touching them with the teat of the bottle: for the instinct of suction is thus aroused. Having in this way, as far as possible, filled the stomach, produce vomiting by tickling the back of the throat with a feather; be careful to keep the material vomited for the doctor, whose successful treatment of the case may depend on this; for on recognising what is the poison he can apply the appropriate antidote. Till his arrival, continue the alternate filling and emptying of the stomach as above described, in order, as far as possible, to remove the poisonous material.

### II. Dangers from Carelessness.

(*a*) **Suffocation in Infants** may be caused in two ways, by stoppage of the mouth and nostrils, or by prevention of the alternate expansion and contraction of the chest, associated with respiration. The former may occur from the face coming in contact with any impervious substance, that limits the entrance of air into the air-passages, as, for instance, a fall into mud or sand, or the presence of thick woollen clothing, cotton-wool or feathers near the face. The latter is due to compression of the chest by too tight clothing, or by some weight acting upon it. Suffocation in either of these ways takes place very readily in young infants, so much so, that it may occur during sleep without any signs of distress on the part of the sufferer. This occurs constantly in cases where young children are overlaid during the night by careless mothers; it has been a great source of infant mortality from the time of King Solomon's wise judgment in such a case to the present day. Another source of danger is the domestic cat, which has so strong a predilection for warm and comfortable resting-places. Such a one is found on the baby's chest while it is asleep in its cot. The soft-footed puss does not wake the child in establishing itself on the gently rising and falling bosom, but its weight gradually wearies out the child's respiratory muscles, which finally cease to contract; breathing ceases, and soon after life is extinct.

**The Symptoms of Suffocation.** In after life, suffocation is accompanied by violent struggling for breath, similar to that which occurs in cases of choking; but in the infant the symptoms may be very slight or altogether absent. In all cases where breathing has ceased, a feather placed near the nostrils does not flutter, a mirror is not dimmed if held in the same position. There is generally a little lividity of the face, more evident in the lips than on the cheeks. The tongue is often pushed up against the front teeth or a little protruded beyond them. The face is placid in patients of this age. Some cause of the occurrence can usually be detected in the infant's surroundings.

**The Treatment of Suffocation.** Remove first anything constricting the chest or neck, or which closes the openings of the mouth or nose, or which blocks up their interior. Incline the head a little forward to relax the air-passages in the neck, the child being laid on its back on a moderately firm pillow. Place the lips in contact with those of the child; close the child's nostrils with the left hand; place the right hand on the front of the child's chest. Blow gently into the mouth, having immediately previously taken a breath.

The child's nostrils being closed, the tendency is for this fresh air to pass down into the lungs; and that it does so will be recognised by the expansion of the chest under the right hand. Having inflated the chest in this way, leave the nose and mouth free, and compress the chest steadily and gently with the right hand. This expels the air previously blown into the lungs, and the natural movements of respiration are thus copied. This form of alternate inspiration and expiration, artificially produced, must be continued patiently and regularly until the arrival of a medical man. It should be done twelve times in each minute. It will frequently result in restoration, even where respiration may have been in abeyance for a considerable time, for in infants the heart will continue to beat for a long time after breathing has ceased. The child's body must be warmed by wrapping in hot blankets, and cloths wrung out of hot water may be advantageously applied over the pit of the stomach, to the calves of the legs, and around the ankles. If natural breathing returns in a feeble way, it must be fortified by watching for the expirations and accompanying each by gentle pressure on the chest. The chest must be left perfectly free at the end of expiration, so that the following inspiration may be as deep as possible. Smelling-salts held near the nose cause increased depth of inspiration, so does cold water dashed on the face; both these should be made use of.

(*b*) **Falls.** Owing to its instinctive tendencies to active movement, an infant may escape from its nurse's arms, or it may be carelessly dropped, and a serious fall may ensue. The most varied results may occur, which cannot be enumerated here. After such a fall, whether the child seems badly hurt or not, keep it very quiet, and have it thoroughly examined by a doctor. There can be little doubt that such a course would have prevented or mitigated many a case of disease or deformity occurring later in life, where the fact of such a fall, that has been thought little of, or concealed by the nurse, is tardily admitted when the information comes too late be of any use in the case.

(*To be continued.*)

